# Prevalence of Pre-Diabetes and Its Associated Risk Factors in Rural Areas of Ningbo, China

**DOI:** 10.3390/ijerph13080808

**Published:** 2016-08-10

**Authors:** Ming Zhao, Hongbo Lin, Yanyan Yuan, Fuyan Wang, Yang Xi, Li Ming Wen, Peng Shen, Shizhong Bu

**Affiliations:** 1 Runliang Diabetes Laboratory, Diabetes Research Center, Ningbo University, 818 Fenghua Road, 315211 Ningbo, China; 1411101261@nbu.edu.cn (M.Z.); 1511101190@nbu.edu.cn (Y.Y.); wangfuyan@nbu.edu.cn (F.W.); xiyang@nbu.edu.cn (Y.X.); 2 Yinzhou District Center for Disease Control and Prevention, 315211 Ningbo, China; lin673160@163.com (H.L.); shen-peng@foxmail.com (P.S.); 3 Shanghai 10th People’s Hospital, University of Tongji, 200240 Shanghai, China; liming.wen@sydney.edu.au; 4 School of Public Health, Sydney Medical School, University of Sydney, 2006 Sydney, Australia

**Keywords:** pre-diabetes, risk factor, prevalence, rural area, interaction

## Abstract

*Objective*: The aims of the study were to investigate the prevalence of pre-diabetes and explore its associated risk factors in rural areas of Ningbo, China. *Methods*: A cross-sectional survey was conducted with 4583 adult residents in rural areas of Ningbo, China between March and May 2013. The survey used a multi-stage, stratified, cluster sampling method. Data collected included demographics and medical history, anthropometric measurements, blood pressure, blood lipid, and plasma glucose. After at least 10 h of overnight fasting, participants underwent an oral glucose tolerance test (OGTT) to identify pre-diabetes. Univariate and multivariate logistic regression analyses were used to evaluate the associated risk factors for pre-diabetes, and to estimate the effect of interaction between the factors. *Results*: There were 1307 survey participants having pre-diabetes (28.52%) and the age-standardized prevalence was 30.53%. Multivariate logistic regression results showed that overweight/obesity, hypertension, and higher triglycerides were the risk factors for developing pre-diabetes. There were positive interactions between overweight/obesity and triglycerides, and also between hypertension and triglycerides on the multiplicative scale, suggesting that they synergistically influenced the development of pre-diabetes. *Conclusions*: The rural areas in Ningbo had a high prevalence of pre-diabetes. Overweight and obesity, hypertension, and elevated triglycerides were the major risk factors. There is a need of early intervention for preventing pre-diabetes.

## 1. Introduction

Diabetes and its chronic complications have become serious public health problems, along with rapid economic development, increase in life expectancy, and changes of lifestyle. The International Diabetes Federation estimated that there were 415 million people with diabetes, and the number is expected to reach 642 million in 2040 [1]. The number of diabetic patients in China is the highest in the world, as diabetes affects about 101 million individuals among people aged between 20 and 79 years, and the number is expected to reach 151 million in 2040 [1].

Pre-diabetes, also known as impaired glucose regulation (IGR), refers to the condition that blood glucose is not as high as in diabetes but is higher than the normal level. It includes impaired fasting glucose (IFG), impaired glucose tolerance (IGT), and IFG combined with IGT [2]. Currently, the population with pre-diabetes has reached approximately 318 million around the world, accounting for 6.7% of the total number of adults. About 69.2% of the pre-diabetes population live in low- or middle-income countries [1]. People with pre-diabetes are at a high risk for developing diabetes, and the prevalence of pre-diabetes has sharply increased in some developing countries in the past decades, especially in China. Two large-scale national surveys conducted in China in 2007 and 2010 showed that the prevalence of diabetes increased from 8.2% to 10.3%, and that of pre-diabetes increased from 16.0% to 50.9% in rural areas, respectively [3,4]. The surveys also found that economically underdeveloped regions had higher prevalence of pre-diabetes than other areas [3,4]. Since pre-diabetes is a transitional stage between normal and diabetes, and it is a reversible process [5], early screening of pre-diabetes is of great significance for early detection and reducing the incidence of diabetes.

Previous cross-sectional studies have reported that multiple risk factors are related to pre-diabetes, such as increased age, overweight, obesity, blood pressure, and dyslipidemia [6,7,8]. More importantly, impaired glucose tolerance was found to be an independent risk factor for cardiovascular disease, the hazard ratio of death was 2.219 (95% CI = 1.076–4.577), and arterial stiffness and pathological changes in the arterial intima occurred in the stage of IGT [9]. However, no epidemiological studies had reported the risk factors for pre-diabetes in rural areas of Ningbo, China.

The purposes of this study were to investigate the prevalence of pre-diabetes and explore its risk factors in rural areas of Ningbo, China, in order to provide evidence for the need of early intervention for pre-diabetes.

## 2. Materials and Methods

### 2.1. Study Design

A cross-sectional survey was conducted with residents aged between 20 to 80 years in rural areas of Yinzhou District, Ningbo, China between March and May 2013. The study was approved by the Ethics Committee of School of Medicine, Ningbo University (project identification code: HEWC-2013-27). All procedures were in accordance with the ethical standards of Helsinki Declaration and all participants provided written informed consent before entering the study.

### 2.2. Context of the Study Setting

Ningbo city is part of the Zhejiang province, located in the southeast coast of China and south of the Yangtze River. It is the second largest city in Zhejiang province, with a total area of 9816 km^2^ and a population of 7.82 million; it has six districts, three county-level cities, and two counties. Among the six districts, Yinzhou District has the largest population in Ningbo, and has 1.35 million inhabitants with 51.28% males and 48.72% females.

### 2.3. Study Participants and Recruitment

A multi-stage, cluster sampling method was used. First, two townships (Jiangshan and Hengxi) were to randomly selected from the total of 17 townships from Yinzhou District. Second, four administrative villages were selected randomly from each of the two townships, respectively. Third, a total of 4682 adult residents who were aged between 20 to 80 years and had lived in the villages for more than six months were selected to participate in this study. Residents with serious diseases and pregnant females were excluded from this study.

### 2.4. Survey Instrument and Survey

A structured questionnaire was designed based on the “Basic schema and data standard of health record (trial)” [10], 2009, and the “National surveillance of non-communicable disease and its risk factors among adults in China” [11], 2010. It covered demographic information including sex, age, education level, and marital status, diet, physical activity, tobacco and alcohol use, and personal health history. The questionnaire interviews were conducted face-to-face by trained investigators, who were staff members of Yinzhou District Center for Disease Control and Prevention between March and May 2013. Current smoking was defined as having smoked 100 cigarettes in one’s lifetime and was still smoking during the study. Current drinking was defined as alcohol intake at least once per month during the previous year [4]. Physical activity was divided into three types according to the frequency of activity in the past 30 days: less than one day a week, 1–4 days a week, or 5–7 days a week [11]. Diet status was also divided into three types, vegetable-based diet, meat-based diet, and meat and vegetable balanced diet. Vegetable-based diet and meat-based diet were defined as vegetable intake and meat intake ≥ 3 times a day, respectively.

### 2.5. Anthropometric and Clinical Measurements

Anthropometric measurements consisted of height and weight. Height and weight were measured to the nearest millimeter and kilogram with the participants standing upright without shoes or thick clothes. Each measurement was completed by two trained investigators, with one investigator taking the measurement and the other recording the reading. Body mass index (BMI) was calculated as the ratio of weight to the square of height (kg/m^2^).

Blood pressure was measured three times with 5 min intervals in a sitting position. Systolic blood pressure (SBP) and diastolic blood pressure (DBP) were measured from the participants’ right arm brachial artery using a mercury sphygmobolometer. The mean values from the three measurements were used for further analysis. The clinical staff members in the Health Service Center of each of township completed the measurements with a standard protocol.

All of the participants were requested to fast overnight for 10 h, and then 2 mL venous blood was collected into vacuum tubes containing sodium fluoride for fasting plasma glucose (FPG) measurement. Participants without a history of diabetes were then given a standard 75-g glucose solution for oral glucose tolerance test (OGTT) [3]. Blood was drawn after 120 min to measure plasma glucose (2h-PG). Blood lipids, including total cholesterol (TC), triglyceride (TG), high-density lipoprotein (HDL), and low-density lipoprotein (LDL), were also measured. All of the blood samples were stored at −20 °C until they were transported to a central, certified laboratory for blood glucose and blood lipid analyses.

### 2.6. Definition of Hypertension, Hyperlipidemia, or Obesity

The definition of hypertension was based on the guidelines formulated by the World Health Organization (WHO). Hypertension was defined as a SBP ≥ 140 mmHg and/or DBP ≥ 90 mmHg, a self-reported history of hypertension, or the use of any anti-hypertension drugs in the past two weeks [12]. Hyperlipidemia was defined as TC ≥ 5.72 mmol/L and/or TG ≥ 1.7 mmol/L, self-reported hyperlipidemia or undergoing treatment [13]. Participants with BMI ≥ 28 kg/m^2^ were considered obese, and those with BMI ≥ 24 kg/m^2^ and < 28 kg/m^2^ were considered overweight [10].

### 2.7. Diagnostic Criteria

The classification of plasma glucose was based on the WHO guidelines [14]. The type of glucose intolerance was determined based on FPG and OGTT as follows: A FPG < 6.1 mmol/L and 2h-PG < 7.8 mmol/L was classified as normal glucose regulation (NGR). Diabetes mellitus (DM) was defined as FPG ≥ 7.0 mmol/L and/or 2h-PG ≥ 11.1 mmol/L, a self-reported history of DM, or the use of diabetic medication. When the glucose level was between NGR and DM, the participant was considered to have pre-diabetes, which was further divided into three categories: isolated impaired fasting glucose (I-IFG), defined as a FPG 6.1–7.0 mmol/L and 2h-PG < 7.8 mmol/L; isolated impaired glucose tolerance (I-IGT), defined as a FPG < 6.1 mmol/L and 2h-PG 7.8–11.1 mmol/L; and IFG combined with IGT (IFG/IGT), defined as a FPG 6.1–7.0 mmol/L and 2h-PG 7.8–11.1 mmol/L.

### 2.8. Statistical Analysis

EpiData (version 3.1, The EpiData Association, Odense, Denmark) was used to collect data and create the database. Statistic Package for Social Science software release 18.0 (SPSS, Chicago, IL, USA) was used for statistical analyses. All of the categorical variables were described and presented as proportions. Continuous variables were first tested for normality, and if the variables had a normal distribution, they were presented as mean values with standard deviation; if the variables did not have a normal distribution, they were presented as median values with quartiles. There were three age groups (20–40, 41–60, and 61–80), three marital status categories (married, unmarried, and divorced/remarried), three diet status categories (meat-based, vegetable-based, and meat and vegetable balanced), four education levels categories (illiterate/semi-illiterate, primary school, secondary school, and tertiary). Secondary school meant junior high school, and tertiary was senior high school and/or above.

Prevalence rates of pre-diabetes in all participants and by age groups were calculated by the direct standardized method based on the 2010 China population census data. The Chi-square test was used for comparisons of categorical variables between groups. Student’s *t*-test was performed to analyze continuous variables with a normal distribution and rank sum test was performed to analyze continuous variables that did not have a normal distribution. Odds ratios (ORs) with 95% confidence intervals (CIs) were calculated using multivariate logistic regression to evaluate the risk factors of pre-diabetes. In multivariate logistic regression, a forward stepwise method was used to enter only the variables with a *p* < 0.2 in the univariate logistic regression. In addition, in the logistic regression, the participants who had normal glucose were regarded as the control group to be compared with those with pre-diabetes.

In order to analyze the interaction between factors in the additive model, an approach raised by Hosmer and Lemeshow was employed [15]. OR_11_ (abbreviated as A) represented the effect of exposed to two risk factors at the same time. When only exposed to one risk factor, the effect was represented as OR_10_ or OR_01_ (abbreviated as B and C, respectively). Three parameters were used to quantify the additive interaction between every two risk factors: the relative excess risk of interaction (*RERI*), the attributable proportion of interaction (*AP*) and the synergy index (*S*) [16]. The formulas are as follows:

*RERI* = A − (B + C − 1)

*AP* = [A − (B + C − 1)]/A

*S* = (A − 1)/[(B − 1) + (C − 1)]

*RERI* indicated the value of interaction between two factors, and the larger the value, the stronger the interaction between the two factors; *AP* represented the ratio of the effect induced by the interaction between the two factors to the total effect. If *RERI* and *AP* equaled 0, or *S* was 1, there was no additive interaction between the factors. We used logistic regression to calculate the regression coefficient and covariance matrix, and then put them into the Excel (Microsoft, Redmond, WA, USA) sheet created by Andersson et al. [17] to analyze the interaction of risk factors and their 95% CIs.

Logistic regression analysis was also used to estimate the interaction on a multiplicative scale. Every two factors and their interaction term were included in the logistic regression model based on the results of multivariate logistic regression analysis. A *p-*value < 0.05 was considered statistically significant.

## 3. Results

### 3.1. Characteristics of the Study Population

A total of 4682 participants were enrolled in this study and 4583 people completed the questionnaire and the examination, giving a respond rate of 97.89%. Table 1 shows the characteristics of the participants stratified by sex. The median age of males recruited in this study was higher than that of females (*p* = 0.004). A majority of the variables differed significantly between males and females, such as marital status, education level, tobacco and alcohol consumption, physical activity, and diet (*p* < 0.05). Based on the BMI data males were 33.6% were overweight and 5.8% were obese, compared with females being 31.0% overweight and 8.7% obese, which were significantly different (*p* = 0.001). However, there were no significant differences in physical activity, hypertension, or SBP between males and females.

### 3.2. Prevalence of Pre-Diabetes

Among the 4583 survey participants, 696 had diabetes, including those who were previously or newly diagnosed. The number of people with pre-diabetes was 1307, giving a crude prevalence of 28.52%, and the age-standardized prevalence was 30.53%. Among all participants with pre-diabetes, 548 were males with an age-standardized prevalence of 35.15% (crude prevalence was 30.42%) and 723 were females with an age-standardized prevalence of 26.10% (crude prevalence was 27.15%). Pre-diabetes was more prevalent in males than females (*p* = 0.016) (Table 2).

### 3.3. Factors Associated with Pre-Diabetes

In order to identify the factors associated with pre-diabetes, we performed univariate and multivariate logistic regression analyses. We found that age (41–60 age group), physical activity, diet, BMI, hypertension, TG, TC, and LDL were associated with pre-diabetes. There were no statistically significant differences in sex, marital status, education level, tobacco or alcohol consumption, or HDL between the pre-diabetes and normal groups (Table 3). The multivariate logistic regression results indicated that overweight (adjusted odds ratio (*AOR* = 1.281, 95% CI = 1.040–1.577), obesity (*AOR* = 1.747, 95% CI = 1.224–2.495), hypertension (*AOR* = 1.286, 95% CI = 1.036–1.597), elevated TG (*AOR* = 1.116, 95% CI = 1.025–1.215) were the independent risk factors of pre-diabetes after adjusting for age, sex, education levels, smoking, physical activity, diet status, TC, LDL-C, and HDL-C (Table 4).

### 3.4. Interaction Analysis of Risk Factors

Subsequently, we used multinomial logistic regression to calculate the regression coefficient and covariance matrix of the factors identified in the multivariate logistic regression analysis after adjustment. The adjusted factors were all the mentioned variables except for BMI, hypertension, and TG. Because the number of obese subjects was relatively small, BMI was divided into two groups, overweight/obesity (BMI ≥ 24 kg/m^2^) and normal (BMI < 24 kg/m^2^). We then examined the additive interaction between every two factors (Table 5). We found no additive interaction between overweight/obesity and hypertension, between overweight/obesity and TG, or between hypertension and TG.

According to pairing between the risk factors, we analyzed the interaction in the multiplicative model between overweight/obesity and hypertension, overweight/obesity and TG, and hypertension and TG (Table 5). The interactions between overweight/obesity and TG, and between hypertension and TG were significant on multiplicative scales (*AO*R = 1.228, 95% CI = 1.023–1.473, and *AOR* = 1.156, 95% CI = 1.002–1.332, respectively). However, no interaction between overweight/obesity and hypertension indicated no statistically significant association with the development of pre-diabetes.

## 4. Discussion

In the current study, the age-standardized prevalence in rural areas in Ningbo was 30.53%, which was lower than that reported in a large-scale epidemiological survey from 31 provinces and municipalities in China conducted in 2010 (50.9%) [4], and it was also lower than the age-adjusted standardized prevalence of pre-diabetes in Chengdu (34.5%) [18]. However, the prevalence in Ningbo was higher than that in Shandong province (6.0%) [19], and that in Hangzhou, another city of Zhejiang province (10.50%) [20]. In addition, the prevalence of pre-diabetes in Ningbo was also higher than that in the Southern Cone of Latin America (17.8%) [21], and Florida (7.3%) [22], but was lower than in the United States (33.1%) [23]. The prevalence was higher in males than in females in this study (*p* = 0.016), and this was similar to previous studies conducted in Tianjing city [8] and Liaoning province [24]. The prevalence of pre-diabetes peaked in the age group of 20–40 years (34.53%), and fell in the age group of 41–60 years (25.35%). This was not consistent with a previous study [25] reporting that the prevalence of pre-diabetes peaked in 35–44 years (in males) and 45–54 and 55–64 years (in females) in rural areas in Shanghai. Several reasons could explain this discrepancy. First, the study participants of different studies might have different lifestyles and environments. Second, the sample size of participants aged 20–40 years was relatively small, so the prevalence rates for this age group might not reflect the real status. Third, rural-to-urban migration was a common phenomenon, particularly in younger and stronger males; however, migrant workers were excluded from our study population, which might have led to the prevalence rate of pre-diabetes among those aged 20–40 years being higher than other age groups, especially in males.

The participants with pre-diabetes had higher BMI, SBP, DBP, TC, and LDL than those without pre-diabetes. In the multivariate logistic regression analysis, we found that high BMI, hypertension, and elevated TG were significantly associated with an increased risk of pre-diabetes, which was consistent with the study conducted by Li et al. [7]. Song et al. [26] found that there were interactions between age ≥45 years, obesity, high-fat diet, and low educational level, and they proposed that screening and early intervention in people with a family history of diabetes and obesity was crucial to reduce the risk of diabetes. Surprisingly, old age, high-fat diet, or low educational level was not associated with developing pre-diabetes in the present study. The reasons for these phenomena may be due to the discrepancy of the age structure of the recruited people in this study, with only 7.27% participants below 40 years old, and the retrospective self-reported dietary intake was difficult to quantify nutrient intake accurately.

No additive interactions existed between any two factors of overweight/obesity, elevated TG, and hypertension. The confidence interval for S in the interaction between hypertension and TG was 0.915, approaching 1, and the results of both RERI and AP indicated a positive additive-scale interaction exist between hypertension and TG. There were interactions on a multiplicative scale between overweigh/obesity and TG, and between hypertension and TG. These findings indicate that overweight/obesity and TG, and hypertension and TG acted synergistically to influence the development of pre-diabetes.

Previous studies have reported that overweight and obesity were the mainly factors contributing to insulin resistance, and insulin resistance was the basis of diabetes and other chronic diseases [13,27]. In the present study, BMI was classified into three types based on Chinese criteria, which was more suitable to the real situation of Chinese people. The total numbers of overweight and obese people in the pre-diabetes and normal groups were 560 and 876, respectively (the total number were 1307 and 2580, respectively), and there were statistically significant differences in being overweight or obese between the pre-diabetes and normal groups (*OR* = 1.354, 95% CI = 1.170–1.567, *OR* = 2.008, 95% CI = 1.540–2.619, respectively) (Table 3). Increasing evidence suggests that the excess body fat in overweight/obese people might lead to increased degradation of fat, which resulted in the production of large amounts of free fatty acids (FFAs). When the level of FFAs was higher in blood, the capacity of liver tissue for insulin-mediated glucose uptake and utilization was lower, so the blood glucose level was high in circulation [28]. In other words, high FFAs in the blood was one of the important pathogenic factors of obesity caused by insulin resistance [29].

A high level of triglycerides was also a risk factor for developing pre-diabetes (*AOR* = 1.116, 95% CI = 1.025–1.215), probably because it could increase the fat deposition in muscle, liver, and pancreas, and it could damage the function of mitochondria and induce oxidative stress which, in turn, could cause insulin resistance, but also lead to impaired islet B cell function [30]. Some studies suggested an interrelation between hypertriglyceridemia and insulin resistance and that they promote each other’s development [31,32]. In some epidemiological studies, for instance, the Framingham Heart Study, hypertriglyceridemia was more prevalent in type 2 diabetes mellitus patients than in the normal population, suggesting that hypertriglyceridemia is a causal factor of type 2 diabetes mellitus [33]. However, this paper was a cross-sectional study, thus it was impossible to determine the causal relationship between hypertriglyceridemia and pre-diabetes.

Hypertension was also found to be a risk factor in our study (*AOR* = 1.286, 95% CI = 1.036–1.597). A possible mechanism is that the activity of angiotensin II is increased in the circulatory system of patient with hypertension. Angiotensin II activates renin-angiotensin-aldosterone system (RAAS) and affects the function of the pancreatic islets, resulting in islet fibrosis and reduced synthesis of insulin, and ultimately leading to insulin resistance [34,35]. Insulin resistance can also aggravate the condition of hypertension. Directly or indirectly through the activity of RAAS, insulin promotes renal tubular to reabsorb Na+ and water, leading to the increased blood volume and cardiac output; this is considered as one of reasons for the development of hypertension [36]. Interactions between abnormal glucose tolerance, hypertension, and dyslipidemia could impair endothelial cell and result in atherosclerosis or other cardiovascular complications. Therefore, the management of daily diet of people with pre-diabetes and the monitoring of body weight, blood lipids, and blood pressure is very important.

A previous study had reported that the progression rate from IFG to diabetes was approximately 8.8% per year [37]. Thus, the high prevalence of pre-diabetes could be a significant risk factor in the development of diabetes. Both diabetes and pre-diabetes (IFG and IGT) are associated with increased risks of cardiovascular disease, which is the major cause of death among Chinese adults [38,39]. In the early stage of IGT, interventions may reverse the pathophysiological changes induced by pre-diabetes, therefore preventing the development of vascular complications associated with hyperglycemia [40]. The Chinese government has allocated a great deal of medical resources for chronic disease prevention, as prevention and early treatment of diseases can release the burdens of chronic diseases on healthcare services.

To our knowledge, this was the first study of exploring the risk factors associated with pre-diabetes in a large rural population in Ningbo, China. All the investigators and staff members involved in the study have undergone a strict training, and all procedures were in accordance with the standard protocol and measurement instructions, which ensure the high quality of data collection. In addition, the questionnaire used in the study has been widely used by various studies in China with good validity and reliability.

In addition to the limitations discussed regarding sample selection there were several more limitations to this study. First, the occupation information of study participants was not collected in our study. As this survey was conducted in rural areas, the majority of the participants were farmers, and the proportion of other types of occupation was small. Second, glycosylated hemoglobin (HbA1c) was not used to detect and diagnose pre-diabetes or diabetes in this study. Only FPG and OGTT were preformed, because these are traditional diagnostic methods used in the region. Third, it was a cross-sectional survey and cannot determine the causal relationship between the studied factors and pre-diabetes; a prospective study is needed to investigate the relationships further.

## 5. Conclusions

In conclusion, this study showed that the age-standardized prevalence of pre-diabetes was 30.53% in rural areas of Ningbo, China. This result indicates that Ningbo will face a high incidence of diabetes in the near future, which will create a heavy economic burden on healthcare. In addition, people who are overweight or obese, or have hypertension or elevated triglycerides should be the focus of diabetes prevention. Health education should be strengthened to encourage people to change unhealthy dietary habits, and regular screening should be performed to reduce the occurrence of new cases of diabetes.

## Figures and Tables

**Table 1:** Characteristics of the study participants stratified by sex in rural areas of Ningbo, China between March and May 2013.

Variable	Male	Female	*p*
Age ^†^	60.0 (50.0–67.0)	58.0 (50.0–65.0)	0.004
Marital Status			
Married	1595 (83.5)	2291 (86.4)	<0.001
Unmarried	250 (13.1)	166 (6.3)	
Divorced/Remarried	64 (3.4)	193 (7.3)	
Education			
Illiterate/semi–illiterate	40 (2.1)	169 (6.4)	<0.001
Primary school	1053 (55.4)	1679 (63.7)	
Secondary school	669 (35.2)	686 (26.0)	
Tertiary	140 (7.3)	103 (3.9)	
Smoking			
Non/Ex–smoker	937 (50.1)	2492 (96.1)	<0.001
Current smoker	933 (49.9)	101 (3.9)	
Alcohol			
Non/Ex–drinker	912 (49.0)	2027 (78.3)	<0.001
Current drinker	951 (51.0)	563 (21.7)	
Physical activity			
Less than 1 day/week	699 (37.7)	944 (36.7)	0.095
1–4 days/week	445 (24.0)	690 (26.8)	
5–7 days/week	711 (38.3)	937 (36.5)	
Diet			
Meat–based	124 (6.6)	121 (4.7)	<0.001
Vegetable–based	31 (1.6)	147 (5.6)	
Meat and vegetables balanced	1727 (91.8)	2338 (89.7)	
Hypertension			
No	603 (34.8)	834 (35.2)	0.751
Yes	1132 (65.2)	1533 (64.8)	
BMI			
<24	1155 (60.6)	1585 (60.3)	0.001
24–28	640 (33.6)	813 (31.0)	
≥28	110 (5.8)	228 (8.7)	
Height ^†^	1.7 (1.6–1.7)	1.6 (1.5–1.6)	<0.001
Weight ^†^	65.0 (58.6–71.0)	57.0 (51.6–62.1)	<0.001
SBP ^†^	136.0 (124.0–148.0)	136.0 (123.0–148.0)	0.436
DBP ^†^	83.0 (76.0–90.0)	81.0 (74.0–88.0)	<0.001
TG ^†^	1.2 (0.9–1.7)	1.4 (1.0–2.0)	<0.001
TC ^†^	4.7 (4.2–5.3)	5.0 (4.4–5.6)	<0.001
LDL-C ^†^	2.8 ± 0.8	3.0 (2.5–3.5)	<0.001
HDL-C ^†^	1.2 (1.0–1.4)	1.3 (1.1–1.4)	<0.001

The column subtotals did not always equal the total because of missing data. ^†^ Continuous variables. Continuous variables were tested for normality, and they were presented as mean ± standard deviation (if they were normally distributed) or presented as median ± quartiles (if they were not normally distributed). BMI, body mass index; SBP, systolic blood pressure; DBP, diastolic blood pressure; TG, triglyceride; TC, total cholesterol; LDL-C, low-density lipoprotein cholesterol; HDL-C, high-density lipoprotein cholesterol.

**Table 2:** The age-standardized prevalence of pre-diabetes stratified by sex and age.

Age Group	Total		Male		Female	
	*n*	%	*n*	%	*n*	%
20–40	115	34.53	72	43.11	43	25.90
41–60	560	25.35	220	26.63	340	24.58
61–80	632	30.97	292	31.5	340	30.52
Total	1307	28.52	584	30.42	723	27.15
Standardized prevalence (%)	30.53		35.15		26.1	

**Table 3:** Factors associated with pre-diabetes in univariate logistic regression analysis.

Variable	β	S.E.	Wald χ^2^	OR (95% CI)	*p*
Age					
20–40					
41–60	−0.328	0.127	6.715	0.720 (0.562–0.923)	0.010
61–80	0.145	0.127	1.299	1.156 (0.901–1.483)	0.254
Sex					
Male					
Female	−0.118	0.068	2.987	0.888 (0.777–1.016)	0.084
Marital Status					
Married					
Unmarried	0.162	0.113	2.063	1.176 (0.943–1.467)	0.151
Divorce/Remarried	0.213	0.148	2.060	1.237 (0.925–1.655)	0.151
Education					
Illiterate/semi-illiterate					
Primary	−0.285	0.166	2.935	0.752 (0.543–1.042)	0.087
Secondary	−0.227	0.171	1.754	0.797 (0.570–1.115)	0.185
Tertiary	−0.012	0.211	0.003	0.988 (0.653–1.495)	0.955
Smoking					
Non/Ex-smoker					
Current smoker	0.140	0.080	3.033	1.150 (0.983–1.347)	0.082
Alcohol					
Non/Ex-drinker					
Current drinker	0.099	0.072	1.862	1.104 (0.958–1.272)	0.172
Physical activity					
Less than 1 day/week					
1–4 days/week	−0.022	0.087	0.064	0.978 (0.824–1.161)	0.801
5–7 days/week	−0.431	0.081	28.384	0.650 (0.554–0.761)	<0.001
Diet					
Meat-based					
Vegetable-based	0.616	0.221	7.765	1.851 (1.200–2.855)	0.005
Meat and vegetables Balanced	−0.257	0.148	3.028	0.773 (0.579–1.033)	0.082
BMI					
<24					
24–28	0.303	0.074	16.580	1.354 (1.170–1.567)	<0.001
≥28	0.697	0.135	26.499	2.008 (1.540–2.619)	<0.001
Hypertension					
No					
Yes	0.405	0.075	29.172	1.499 (1.294–1.736)	<0.001
Height ^†^	−1.325	0.323	16.775	0.266 (0.141–0.501)	<0.001
Weight ^†^	0.012	0.004	12.321	1.012 (1.005–1.020)	<0.001
SBP ^†^	0.015	0.002	53.665	1.015 (1.011–1.019)	<0.001
DBP ^†^	0.013	0.003	13.921	1.013 (1.006–1.020)	<0.001
TG ^†^	0.207	0.037	31.283	1.230 (1.144–1.323)	<0.001
TC ^†^	0.174	0.038	20.823	1.190 (1.105–1.283)	<0.001
LDL-C ^†^	0.116	0.057	4.134	1.123 (1.004–1.256)	0.042
HDL-C ^†^	−0.305	0.157	3.805	0.737 (0.542–1.001)	0.051

S.E., standard error; OR, odds ratio; CI, confidence interval; ^†^ Continuous variables; BMI, body mass index; SBP, systolic blood pressure; DBP, diastolic blood pressure; TG, triglyceride; TC, total cholesterol; LDL-C, low-density lipoprotein cholesterol; HDL-C, high-density lipoprotein cholesterol.

**Table 4:** Factors associated with pre-diabetes in multivariate logistic regression analysis.

Variable	Crude OR (95% CI)	*p*	AOR *(95% CI)	*p*
BMI				
<24				
24–28	1.249 (1.067–1.462)	0.006	1.281 (1.040–1.577)	0.020
≥28	1.777 (1.340–2.358)	<0.001	1.747 (1.224–2.495)	0.002
Hypertension				
No				
Yes	1.381 (1.186–1.607)	<0.001	1.286 (1.036–1.597)	0.023
TG ^†^	1.154 (1.075–1.240)	<0.001	1.116 (1.025–1.215)	0.012

OR, odds ratio; AOR, adjusted odds ratio; CI, confidence interval; BMI, body mass index; TG, triglyceride; * Adjusted for age, sex, marital status, education levels, smoking, drinking, physical activity, diet status, total cholesterol, low-density lipoprotein cholesterol, and high-density lipoprotein cholesterol; ^†^ TG was presented as a continuous variable.

**Table 5:** Interaction of risk factors for pre-diabetes.

Factor 1	Factor 2	Additive Interaction				Multiplicative Interaction	
		OR (95% CI)	*RERI* (95% CI)	*AP* (95% CI)	*S* (95% CI)	AOR * (95% CI)	*p*
Overweight/Obesity	Hypertension						
+	+	2.009 (1.655–2.439)	0.248 (−0.203–0.700)	0.124 (−0.096–0.343)	1.326 (0.749–2.348)	1.262 (0.840–1.897)	0.262
+	−	1.360 (1.056–1.751)					
−	+	1.403 (1.162–1.693)					
−	−	1					
Overweight/Obesity	TG ^‡^						
+	+	2.005 (1.641–2.451)	0.403 (−0.046–0.853)	0.201 (−0.005–0.407)	1.669 (0.875–3.183)	1.228 (1.023–1.473)	0.027
+	−	1.335 (1.117–1.595)					
−	+	1.268 (1.027–1.565)					
−	−	1					
Hypertension	TG ^‡^						
+	+	2.034 (1.640–2.522)	0.602 (0.146–1.058)	0.296 (0.091–0.501)	2.394 (0.915–6.264)	1.156 (1.002–1.332)	0.047
+	−	1.331 (1.097–1.614)					
−	+	1.101 (0.820–1.477)					
−	−	1					

OR, odds ratio; AOR, adjusted odds ratio; CI, confidence interval; RERI, relative excess risk of interaction; AP, attributable proportion of interaction; S, synergy index; TG, triglyceride; BMI, body mass index; normal: BMI < 24 kg/m^2^; overweight/obesity: BMI ≥ 24 kg/m^2^; * Adjusted for age, sex, marital status, education level, smoking, drinking, physical activity, diet, total cholesterol, low-density lipoprotein cholesterol, and high-density lipoprotein cholesterol; ^‡^ In the additive model, TG was presented as a categorical variable; in the multiplicative model, TG was presented as a continuous variable.
